# Thermal Responsiveness of 1,2,4-Triazolium-Based Poly(ionic liquid)s and Their Applications in Dye Extraction and Smart Switch

**DOI:** 10.1021/acsapm.4c02446

**Published:** 2024-10-28

**Authors:** Feng Chen, Jiefeng Zhu, Ruijie Hou, Xianjing Zhou, Jiayin Yuan, Xinping Wang

**Affiliations:** ‡ School of Chemistry and Chemical Engineering, Key Laboratory of Surface and Interface Science of Polymer Materials of Zhejiang Province, Zhejiang Sci-Tech University, Hangzhou 310018, China; § State Key Laboratory for Modification of Chemical Fibers and Polymer Materials, College of Materials Science and Engineering, Donghua University, Shanghai 201620, China; ⊥ Department of Materials and Environmental Chemistry, Stockholm University, Stockholm 10691, Sweden

**Keywords:** 1,2,4-triazolium, poly(ionic liquid), thermoresponsiveness, dye extraction, smart switch

## Abstract

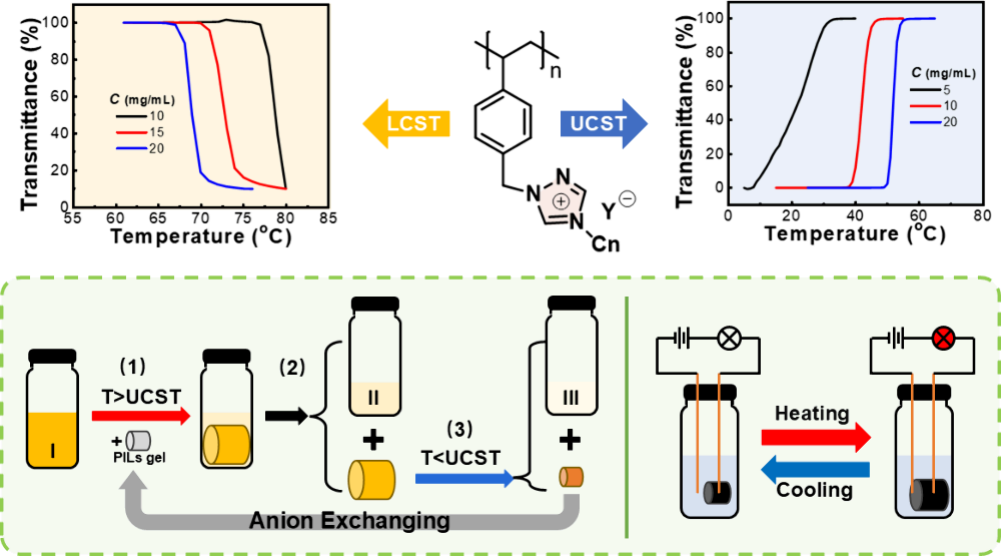

Triazoliums are a family of five-membered heterocyclic cations that contain three nitrogen and two carbon atoms. In contrast to the widely studied imidazolium cations, triazoliums are less explored. In terms of the chemical structure, triazolium replaces a carbon atom in the imidazolium cation ring with an electron-withdrawing nitrogen atom, which makes the triazolium more polarized. Among the many physical properties, the thermal responsiveness of triazoliums is of particular interest to us but has been rarely investigated. In this contribution, we prepared a series of 1,2,4-triazolium-based poly(ionic liquid)s (PILs) with varying alkyl substituents and counteranions and studied their thermal-responsive behavior. We found that 1,2,4-triazolim-based PILs with a polymeric backbone structure similar to that of polyimidazoliums exhibited opposite thermal phase transition processes in solvents. For example, methyl-substituted 1,2,4-triazolium-based PILs exhibited an upper-critical-solution-temperature (UCST)-type phase transition in methanol when the counterion was I^–^ and a lower-critical-solution-temperature (LCST)-type phase transition in acetone when the counterion was PF_6_^–^. The thermal responsiveness was reversible and concentration-dependent. Interestingly, the thermal response of 1,2,4-triazolim-based PILs could be retained in the organogel form, which was applied in the pretreatment of anion-containing organic waste liquids and temperature-controlled “smart” switches.

## 1 Introduction

Stimulus-responsive “smart” polymers are capable of triggering cascading microscopic and macroscopic changes in response to variations in environmental conditions, making them suitable for a multitude of applications ranging from drug delivery and biosensors to tissue engineering and “smart” surfaces.^1,2^ Thermoresponsive systems have been some of the most prevalent in “smart” materials due to their thermal reversibility in solution.^3−5^ Thermoresponsive polymers exhibit a wide range of temperature-driven solution behaviors in water or organic solvents.^6^ Typical thermosensitive polymers are based on polyacrylamides, polycaprolactams, polyethers or poly(acrylic acid), and more.^6^ The best known is the poly(*N*-isopropylacrylamide) (PNIPAM) with a lower critical solution temperature (LCST)-type phase transition behavior in aqueous solution.^7,8^

Poly(ionic liquid)s (termed PILs) are a subclass of polyelectrolytes that have an ionic liquid (IL)-like structure in each repeating unit. They inherit and combine some excellent properties of ILs and polymers and have attracted wide interest in the fields of polymer science, catalysis, separation, sensors, cell biology, and electrochemistry.^9−11^ Much of the research activity on PILs to date has focused on characterizing and understanding their physical and chemical properties in their neat state and their corresponding materials applications.^12^ Lately, there has been increasing interest in exploring PILs in a solution state. The structural diversity of anions and cations of IL monomers greatly facilitates fine-tuning of the hydrophobic/hydrophilic balance of the corresponding PILs, which may exhibit thermal phase transitions in appropriate solvents. Such examples include studies of thermosensitive behaviors of poly(4-tetrabutylphosphonium styrenesulfonate) in aqueous solvents^13−15^ and poly(vinyl ethers) with imidazolium or pyridinium pendants in organic solvents.^16^

Polymers possessing imidazolium cations are by far the most intensively studied PILs. Replacing one carbon atom in the imidazolium ring with a more electron-withdrawing nitrogen atom yields the triazolium cation. This leads to enhanced polarization of the C–H bond in the heterocyclic cation, resulting in stronger Brønsted acidity and hydrogen bonding than in imidazolium,^17^ thus expanding the property window and the application spectrum of PILs. Depending on the relative positions of nitrogen atoms, there are two isomers of the triazolium unit, i.e., 1,2,3- and 1,2,4-triazolium. 1,2,3-Triazolium PILs have been intensively investigated by Drockenmuller^18,19^ and Miller.^20,21^ Recently, our group constructed a series of 1-vinyl-1,2,4-triazolium-type PILs.^17,22,23^ However, several papers have reported the thermal response properties of 1,2,3-triazolium-based PILs.^24^ To the best of our knowledge, there is no report on thermally responsive 1,2,4-triazolium-based PILs and their corresponding applications. Due to increased Lewis acidity, we expect interesting solution behavior of 1,2,4-triazolium PILs different from the 1,2,3-triazolium ones.

In this work, a series of 1,2,4-triazolium-based PILs carrying alkyl substituents of different lengths and different counteranions were designed and synthesized. It was found that such PILs with tailored structures present thermal-responsive behaviors opposite to those of the imidazolium-based PILs. Furthermore, these 1,2,4-triazolium-based thermal-sensitive PILs could be formulated into organogels for applications such as the recycling of dye waste liquids and the manufacture of temperature-controlled switches.

## 2 Experimental Section

### 2.1 Materials

1,2,4-Triazole sodium salt, 4-vinylbenzyl chloride, iodomethane, 1-bromobutane, 1-bromooctane, 1,3-dibromopropane, potassium hexafluorophosphate (KPF_6_), potassium tetrafluoroborate (KBF_4_), 1-methylimidazole, carbon nanotube (CNT), hydroquinone, methyl orange (MO), methyl blue (MB), orange G (OG), bromothymol blue (BTB), and solvents of analytical-reagent grade were purchased commercially and used without further purification. 2,2′-Azobis(2-methylpropionitrile) (AIBN) was recrystallized from methanol before use.

### 2.2 Synthesis of the Neutral Intermediate triaz

4-Vinylbenzyl-1,2,4-triazole (termed “triaz”) was synthesized according to the literature.^25,26^ In a typical run, 1,2,4-triazole sodium salt (5.46 g, 59.3 mmol, 1.17 equiv) and 4-vinylbenzyl chloride (7.75 g, 50.8 mmol, 1 equiv) were dissolved in 250 mL of *N*,*N*-dimethylformamide (DMF). The reaction mixture was then stirred at room temperature for 24 h. After the reaction, an equal volume of deionized water was added to the solution, which was subsequently extracted four times with ethyl acetate. The combined organic layers were concentrated under vacuum and extracted with water four times. The organic layer was dried over anhydrous MgSO_4_, filtered off, and then concentrated under vacuum to give a pale-green liquid (8.5 g, 91.2%).

### 2.3 Synthesis of the Monomer triaz-Cn-X and the Cross-Linker Dtriaz-Br

The 1,2,4-triazolium-based monomer (triaz-Cn-X) and cross-linker (Dtriaz-Br) were synthesized according to the literature.^27,28^ Take triaz-C1-I as an example: triaz (4.7 g, 25 mmol, 1 equiv) and iodomethane (5.4 g, 38 mmol, 1.5 equiv) were dissolved in 10 mL of tetrahydrofuran (THF). Hydroquinone (10 mg) was added to the reaction mixture as a polymerization inhibitor. The reaction mixture was then stirred at 50 °C for 24 h. An excess of THF was then added, and the mixture was stirred for a while to allow the product to completely precipitate out. The yellow solid was collected by filtration and dried at room temperature until constant (5.76 g, 71%). Other triaz-Cn-X and Dtriaz-Br were synthesized in a similar process.

### 2.4 Synthesis of Ptriaz-Cn-X

Poly(4-vinylbenzyl-1,2,4-triazolium) PILs (Ptriaz-Cn-X) were synthesized according to the literature.^29,30^ Take Ptriaz-C1-I as an example: triaz-C1-I (2.5 g, 7.6 mmol, 1 equiv) and AIBN (0.025 g, 0.15 mmol, 0.02 equiv) were dissolved in 10 mL of DMF. The reaction mixture was then stirred at 75 °C under nitrogen for 24 h. The product was purified by dialysis against water. A yellow solid was obtained after freeze-drying (1.03 g, 41.2%).

A total of 1 g of Ptriaz-C1-I was dissolved in 10 mL of water. A total of 2 g of fluorinated salts (KBF_4_ or KPF_6_) was dissolved in 50 mL of water. Then the Ptriaz-C1-I aqueous solution was added to the KBF_4_ or KPF_6_ solution, and the mixture was stirred at room temperature for 24 h. The reaction mixture was then filtered off to give a white solid. The solid was collected and dried under vacuum at 60 °C for 48 h.

### 2.5 Synthesis of 4-Vinylbenzylimidazolium Chloride Monomer and Polymer

1-Methyl-4-vinylbenzylimidazolium chloride monomer (Im-C1-Cl) and its polymer (PIm-C1-Cl) were synthesized according to the literature.^31,32^ Synthetic details are described in the Supporting Information.

### 2.6 Preparation of Ptriaz-C1-I and CNT-PIL Organogels

Triaz-C1-I (1 g, 15 equiv), Dtriaz-Br (0.18 g, 1 equiv), and AIBN (30 mg) were dissolved in 3 mL of DMF. Nitrogen was bubbled through the reaction solution for 30 min to remove oxygen. It was then transferred to a closed mold and reacted at 75 °C for 24 h to obtain the primary PIL organogel. It was then immersed in methanol to displace the liquid phase from DMF to methanol, and a Ptriaz-C1-I gel was thus obtained. The CNT-PIL organogel was prepared by the same procedure, except that 30 mg of CNTs was added initially.

### 2.7 Characterization

^1^H and ^13^C NMR measurements were recorded at 400 MHz on a Bruker spectrometer with an AVANCE II console at different temperatures. Spectra were run with 16 transients and a relaxation delay of 30 s and an acquisition time of 4 s (total running time of approximately 15 min). High-resolution mass spectrometry (HRMS) spectrum was recorded on a Waters TOF-MS GCT Premier instrument using electrospray ionization. Fourier transform infrared (FT-IR) spectra were recorded on a Nicolet iS10 spectrometer. Size-exclusion chromatography (SEC) measurements of PILs were carried out on a Waters 1515 SEC system. DMF (containing 10 mM LiBr) was used as the eluent at a flow rate of 1.0 mL/min. Narrow linear poly(methyl methacrylate) standards were used for calibration. The degree of anion exchange of PILs was quantified by a total organic halogen analyzer (AOX/TOX multi X 2500 analyzer). UV–vis spectra were measured by a Cary300 UV–vis spectrophotometer. Dynamic light scattering (DLS) tests were carried out on a 90Plus particle size analyzer at a scattered light angle of 90° and a laser wavelength of 635 nm.

## 3 Results and Discussion

### 3.1 Synthesis of the Triazolium-Based PILs

**Figure 1:**
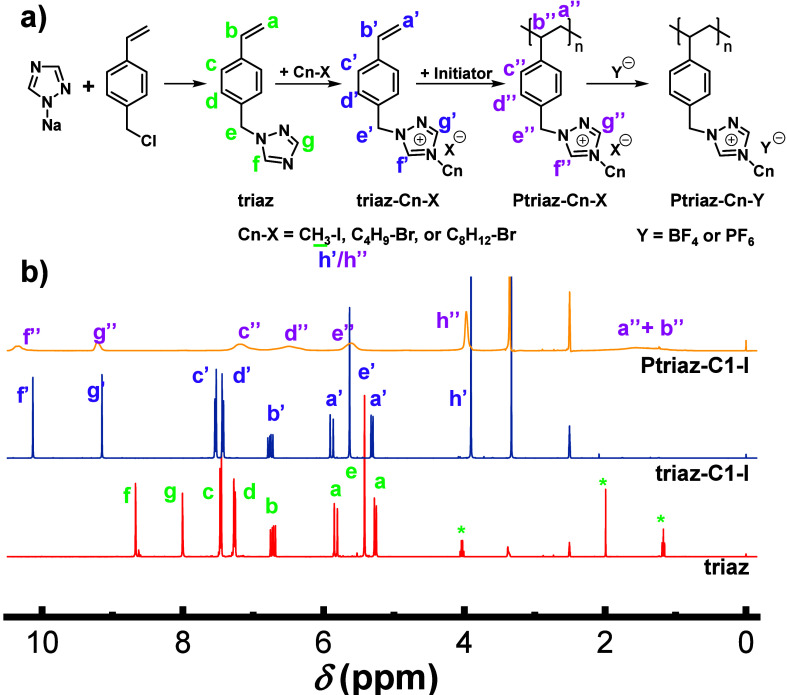
(a) Synthetic route toward 1,2,4-triazolium-based PILs with different alkyl chain lengths and counteranions. (b) ^1^H NMR spectra of the neutral intermediate triaz, the IL monomer triaz-C1-I, and its PIL Ptriaz-C1-I. The solvent is DMSO-*d*_6_. Peaks marked with asterisks are attributed to the solvent residual of ethyl acetate.

To start, we first synthesized a series of 1,2,4-triazolium-based PILs with different alkyl substituents (methyl, *n*-butyl, and *n*-octyl) and counteranions (Br^–^, I^–^, BF_4_^–^, and PF_6_^–^), as shown in Figure 1a. The 4-vinylbenzyl chloride first reacted with the heterocycle of sodium 1,2,4-triazolate, producing 1-(4-vinylbenzyl)-1,2,4-triazole (termed “triaz”). The subsequent N-alkylation of 1,2,4-triazole, its polymerization, and the following anion-exchange reactions were conducted similarly to our previous report.^30,33^ The PIL samples were named Ptriaz-Cn-X, where Ptriaz, Cn, and X denote the poly(1,2,4-triazolium) backbone, the alkyl chain length in terms of the number of carbons, and the counteranions, respectively. Similarly, triaz-Cn-X was used for the monomers. The chemical structure of the samples was characterized by ^1^H NMR, ^13^C NMR, HRMS, FT-IR, and SEC, and the results are shown in Figures 1b and S1–S11. The PIL Ptriaz-C1-I with a methyl substituent and iodide as the counteranion was given here as an example of characterization and assignment of its molecular chemical structure (Figure 1b). In deuterated dimethyl sulfoxide (DMSO-*d*_6_), the chemical shifts of H_f_ and H_g_ in the 1,2,4-triazole moiety of triaz were located at 8.67 and 8.00 ppm, respectively. By contrast, the H_f′_ and H_g′_ peaks in 1,2,4-triazolium moiety of the triaz-C1-I monomer underwent a downfield shift upon quaternization, with their chemical shifts at 10.13 and 9.15 ppm, respectively, suggesting a successful nucleophilic substitution.^34^ As for Ptriaz-C1-I, H_a′_ and H_b′_ attributed to the protons of the double bond in the spectra of the neutral triaz and the triaz-C1-I monomer disappeared completely, while broad peaks emerged at the chemical shifts of 1.40–1.60 ppm, which are assigned to H_a″_ and H_b″_, respectively; all of the signal peaks in Ptriaz-C1-I were broadened, indicating that the polymerization was successfully conducted. The ^13^C NMR and HRMS spectra of triaz-C1-I are shown in Figures S2 and S3, respectively . The ^1^H NMR spectra of the other IL monomers and the corresponding PILs are collected in Figures S4–S7. The apparent molecular weights of the series of PILs, i.e., Ptriaz-C1-X, Ptriaz-C4-X, and Ptriaz-C8-X, were measured by SEC (Figures S8–S10), and the results are listed in Table S1. The occurrence of anion exchange was initially qualitatively confirmed by FT-IR tests, as shown in Figure S11. For Ptriaz-C1-I, because I^–^ is invisible in FT-IR, only three typical bands at 1453, 1585, and 1630 cm^–1^ associated with the stretching vibrations of the 1,2,4-triazolium ring were recorded in the spectrum.^30,35^ For Ptriaz-C1-BF_4_, the band at 1058 cm^–1^ corresponds to the asymmetric stretching vibration of BF_4_^–^.^36^ For Ptriaz-C1-PF_6_, the broad band at 838 cm^–1^ corresponds to the symmetric stretching vibration of PF_6_^–^.^30^ In addition, the degree of anion exchange of PILs was quantified using a total organic halogen analyzer, and all were above 80%, as detailed in Table S1. As a control, we also synthesized the corresponding imidazolium-based PILs (termed PIm-C1-X, where X = Cl or I). Their synthetic routes and structural characterizations are shown in Figures S12–S14.

### 3.2 Thermal Responsiveness of 1,2,4-Triazolium-Based PILs

In 1,2,4-triazolium-based PILs, the polarity of benzyl and triazolium cations was quite different; the polarity of the counteranion could be adjusted by the types of the anions through the anion-exchange reaction.^37^ In this regard, 1,2,4-triazolium-based PILs in the solution can tune the polymer–polymer and polymer–solvent interactions in a wide range.^12^ We examined the solubility of 1,2,4-triazolium-based PILs with different alkyl chain lengths (methyl, *n*-butyl, or *n*-octyl) and counteranions (Br^–^, I^–^, BF_4_^–^, or PF_6_^–^) and compared them with the corresponding imidazolium-based PILs at 25 and 80 °C (Table S2). The solubility of specific 1,2,4-triazolium-based PILs was found to be non-temperature-dependent in most solvents. In addition, the solubility of a PIL in different solvents varies with its structure. For example, the Ptriaz-C1-X samples bearing different counteranions are all soluble in solvents with high dielectric constant such as DMSO (ε = 47.24) and DMF (ε = 36.71), insoluble in solvents with low dielectric constant such as THF (ε = 7.52) and toluene (ε = 2.38), and limitedly soluble in ethanol (ε = 25.3) and isopropyl alcohol (ε = 8.35). Their solubility in water typically decreases with increasing hydrophobicity of counteranions and *vice versa* in acetone. As the alkyl length increases from methyl to *n*-butyl and *n*-octyl, the solubility of PILs decreases in water and gradually increases in organic solvents, e.g., THF, CHCl_3_, and acetonitrile. Interestingly, Ptriaz-C1-I was found to exhibit an upper critical solution temperature (UCST)-type phase transition in methanol, whereas Ptriaz-C1-PF_6_ with the same polymer backbone exhibits a LCST-type phase transition in acetone. In addition, Ptriaz-C4-Br also exhibited the UCST phase transition in isopropyl alcohol. It is notable that imidazolium-based PILs with similar structures did not exhibit the same thermal responsiveness in the 12 solvents tested.

**Figure 2:**
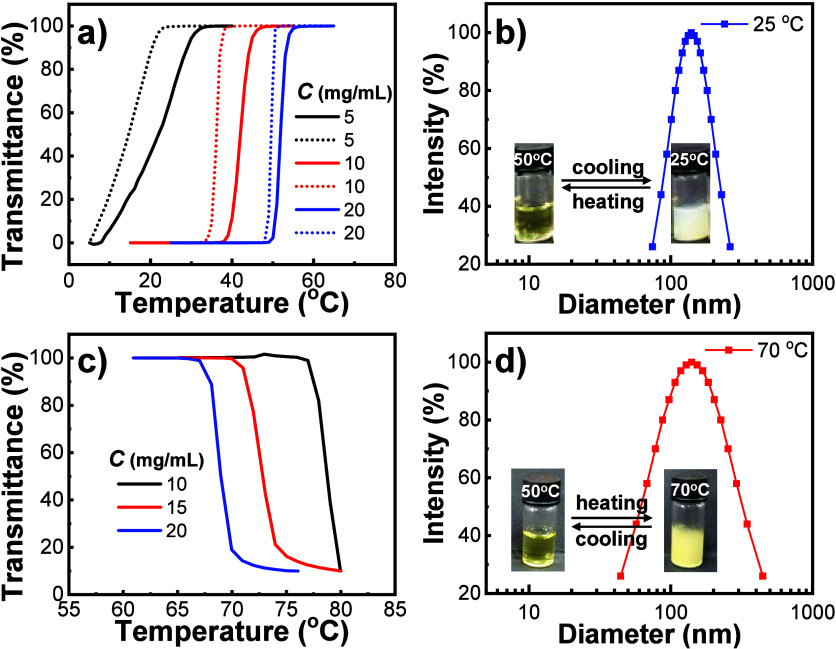
(a and c) Turbidity curves of the Ptriaz-C1-I solution in methanol and the Ptriaz-C1-PF_6_ solution in acetone at various concentrations, respectively. The solid and dashed lines represent heating and cooling processes, respectively. (b and d) Hydrodynamic diameters of particles measured in the Ptriaz-C1-I solution in methanol and the Ptriaz-C1-PF_6_ solution in acetone below and above *T*_c_, respectively. Insets: Photographs of the corresponding solutions.

We next studied the thermal responsiveness of Ptriaz-C1-X. Parts a and c of Figure 2 show that Ptriaz-C1-X with different counteranions, i.e., I^–^ and PF_6_^–^, exhibit UCST- and LCST-type phase transition behaviors in methanol and acetone, respectively. In addition, such thermal-responsive behavior was reversible and concentration-dependent. At elevated temperature, Ptriaz-C1-I gradually changed from aggregates (*d* = 75–250 nm, Figure 2b) to complete dissolution (no signal above 100 nm in DLS tests) in methanol, accompanying the change of the solution from turbid to transparent. The cloud point (*T*_c_, defined as the temperature at 80% transmittance at λ = 550 nm in heating) increased from 27 to 53 °C when the concentration increased from 5 to 20 mg/mL (Figure 2a). Upon cooling, the corresponding *T*_c_ was relatively lower because of the hysteresis of polymer precipitation. In the case of the Ptriaz-C1-PF_6_ solution in acetone, as the temperature increased, the PILs gradually aggregated (*d* = 50–450 nm, Figure 2d). *T*_c_ decreased from 78 to 68 °C when the concentration increased from 10 to 20 mg/mL (Figure 2c). Note that the *T*_c_ values of the Ptriaz-C1-PF_6_ solutions are higher than the boiling point of acetone because of the strong interaction between PILs and solvent.^38^ However, in the cooling process, the temperature of the solution needed to be raised to 80 °C and held for 10 min. During this process, acetone continuously volatilized, which affected the concentration of the solution, so an accurate cooling process cannot be measured in our current setup. In addition, Ptriaz-C4-Br also exhibited a UCST-type phase transition in isopropyl alcohol, as is similarly analyzed in Figures S15 and S16.

**Figure 3:**
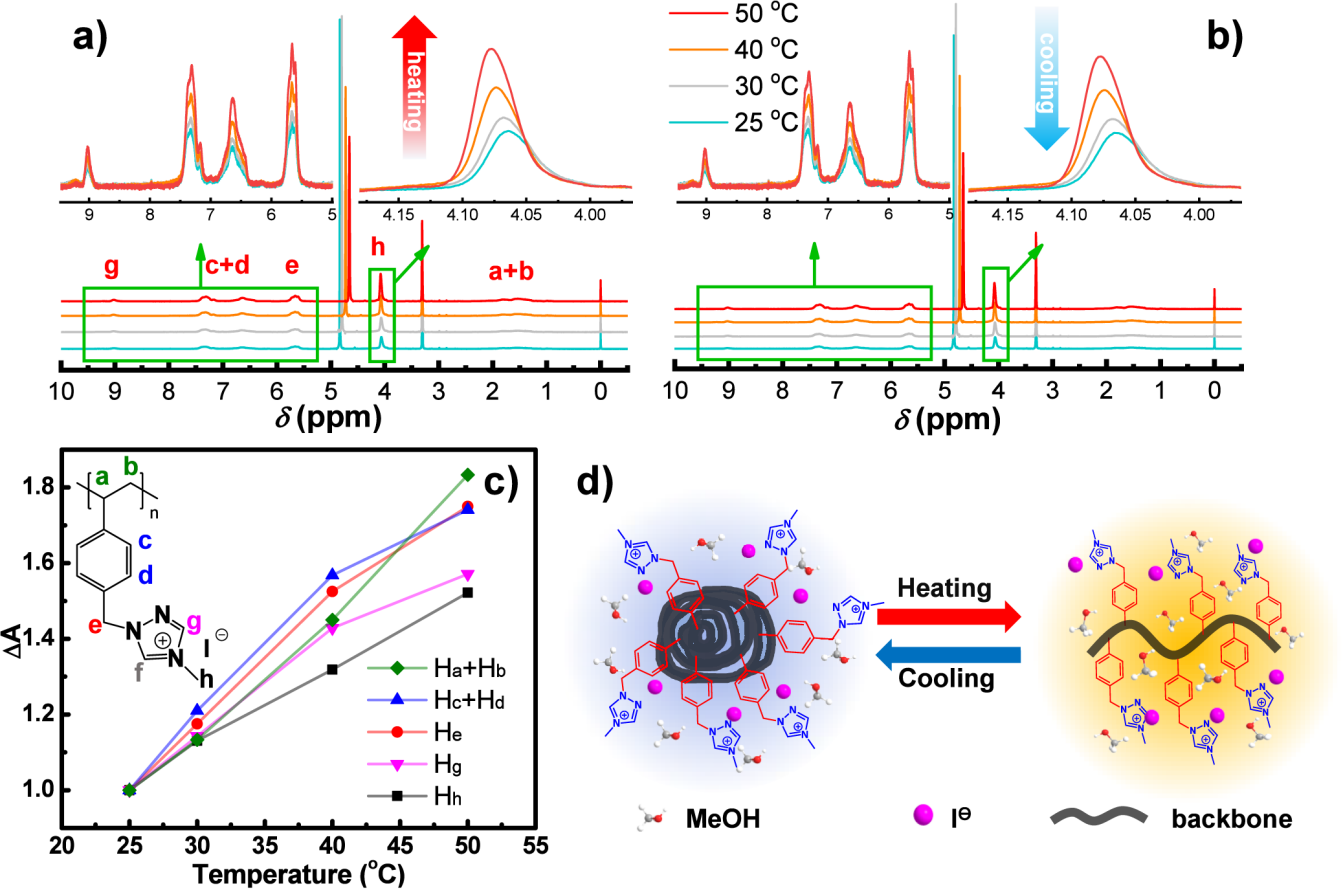
Temperature-variable ^1^H NMR spectra of Ptriaz-C1-I in CD_3_OD: (a) 25 → 50 °C; (b) 50 → 25 °C. The inset shows an enlarged view. The concentration of Ptriaz-C1-I was 10 mg/mL. (c) Corresponding integral area change of different protons (Δ*A*) as a function of the temperature. The integral areas of different protons of Ptriaz-C1-I measured at 25 °C were normalized as 1. (d) Schematic illustration of the dynamic phase transition mechanism of the Ptriaz-C1-I solution in methanol during the heating and cooling processes.

To understand the temperature-dependent phase transition of Ptriaz-C1-I in methanol, temperature-variable ^1^H NMR measurements were performed, as shown in Figure 3a,b. The intensity of almost all proton peaks was found to increase with rising temperature, indicating that Ptriaz-C1-I dissolved better at higher temperature. When the temperature decreased from 50 to 25 °C, the intensity of the proton peaks was reduced expectedly. The integral area of backbone protons (H_a_ and H_b_) and benzyl (H_c_, H_d_, and H_e_) changed more than that of the methyl triazolium protons (H_g_ and H_h_), as shown in Figure 3c, revealing that temperature affects more the solubilization effect of the backbone and the benzyl unit. Note that the integral areas of different protons of Ptriaz-I measured at 25 °C were normalized as 1. In addition, C5–H (i.e., H_f_) of 1,2,4-triazolium is highly polarized^17^ and thus exchanges with the deuterium of CD_3_OD, rendering it invisible in the spectra. Sun et al.^39^ calculated the p*K*_a_ value of 8.62 for C5–H in 1,2,4-triazolium, indicating a relatively strong acidic character, which favors the hydrogen–deuterium exchange.

On the basis of the temperature-dependent ^1^H NMR tests, we have drawn a schematic diagram of the dynamic mechanism of the phase transition of Ptriaz-C1-I in methanol during heating and cooling processes, as shown in Figure 3d. Under the UCST condition, the polymer backbone and the benzyl group are less soluble in methanol than the methyltriazolium cation, and thus Ptriaz-C1-I chains aggregate driven by the solvophobic effect, with the polymer backbone distributed in the core and the methyltriazolium cation in the periphery in contact with solvent molecules. As the temperature increases, the solvent molecules diffuse into the polymer backbone, and the densely assembled structure starts to molecularly dissociate through relaxation of the backbone. At higher concentrations of Ptriaz-C1-I, more energy is required for the solvent molecules to diffuse into and relax in the polymer backbone; thus, the phase transition temperature is higher.^40,41^

### 3.3 Application of Thermosensitive Triazolium-Based PILs Organogels

**Figure 4:**
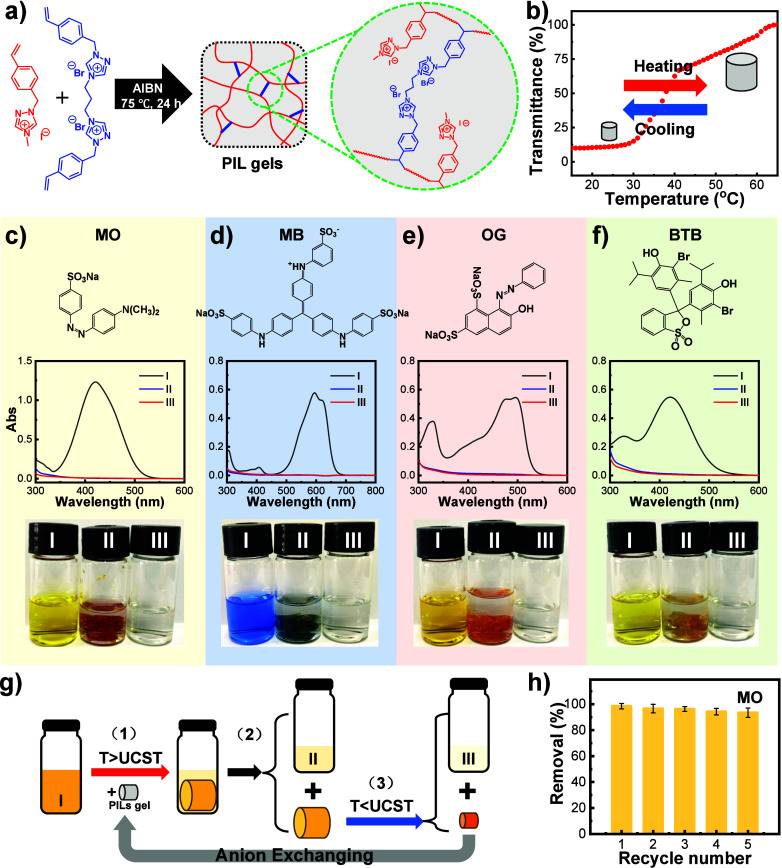
(a) Schematic representation of the synthesis of 1,2,4-triazolium-based PILs organogels. (b) Turbidity curve of the organogels in methanol as a function of the temperature. (c–f) UV–vis spectra and photographs of the dye solution in methanol before and after treatment by the PILs organogel. Solutions I–III represent the initial dye solution, the surrounding solution after addition of the PILs organogel, and the solution squeezed out of the PILs organogel by reversible thermoresponse, respectively. The tested dyes are (c) MO, (d) MB, (e) OG, and (f) BTB, respectively. (g) Schematic diagram of the PILs organogel treatment of the dye solution in methanol. (h) Cyclic removal of MO from its methanol solution by a Ptriaz-C1-I gel.

Organogels are gels in which the organic solvent is immobilized in a network of gel aggregates. They can swell and retain large amounts of organic solvents, absorb and release substances, and respond to a variety of physical and chemical stimuli.^42^ By the design of a gel matrix structure and selection of a suitable liquid phase, organogels can vary their hydrophobicity,^43^ anti-icing,^44^ antiadhesion,^45^ etc. Because Ptriaz-C1-I exhibits a UCST-type phase transition in methanol, we copolymerized its monomer triaz-C1-I with a 1,2,4-triazolium-based cross-linker (termed Dtriaz-Br, structurally characterized in Figure S17) to obtain an organogel (termed Ptriaz-C1-I gel; Figure 4a). The cross-linked Ptriaz-C1-I organogel remained thermoresponsive; i.e., it changed from opaque to transparent with increasing temperature (Figure 4b), accompanied by swelling of the volume.

Because the Ptriaz-C1-I organogel is rich in 1,2,4-triazolium cations, it can be used as an adsorbent to extract organic anions from organic solvents via electrostatic interactions and anion exchange. In combination with its UCST-type phase transition in methanol, the separation and recovery of organic solvents from their waste liquids can be achieved. Four organic anionic dyes (MO, MB, OG, and BTB) were chosen as models, and their molecular structures are shown in Figure 4c–f. The standard curves for the four dyes are shown in Figures S18–S21.

A typical procedure for Ptriaz-C1-I organogel treatment of anionic dyes in their methanol solutions proceeds as follows: (1) The Ptriaz-C1-I organogel was immersed in the dye solution in methanol solution I, and the gel was allowed to fully adsorb the dye at a temperature higher than its UCST (e.g., 60 °C). (2) The gel is removed from the solution, and the remaining solution is denoted as solution II. (3) Because the organogel is thermoresponsive, i.e., swelling at high temperature and deswelling at low temperature, the internal liquid in the organogel will be squeezed out when the organogel is cooled below its UCST, denoted as solution III. A schematic of the whole process is shown in Figure 4g. Solutions I–III before and after treatment were characterized using UV–vis spectroscopy, as shown in Figure 4c–f. The initial MO, OG, and BTB solutions I in methanol were of different shades of yellow, and the initial MB solution I in methanol was blue. All of the dye solutions I have distinct characteristic peaks in the UV–vis spectra. In contrast, after the addition of Ptriaz-C1-I organogel, the anionic dyes were enriched inside the gel due to the electrostatic effect and anion-exchange reaction, whereas solution II outside the gel and solution III squeezed out of the gel were essentially colorless and the UV–vis absorbance was close to zero. Note that, due to the small volume of the gel, solution III squeezed out after cooling of the gel is the sum of several repetitions of experiments. In addition, the gel with adsorbed anionic dyes can be immersed in a KI solution to release the dyes by anion exchange, thus regenerating the gel to be used for the next dye extraction. As an example, the Ptriaz-C1-I gel showed satisfactory dye removal for MO after five cycles of extraction and regeneration (Figure 4h). This work provides an alternative method to treat anionic dye-containing organic waste liquids.

**Figure 5:**
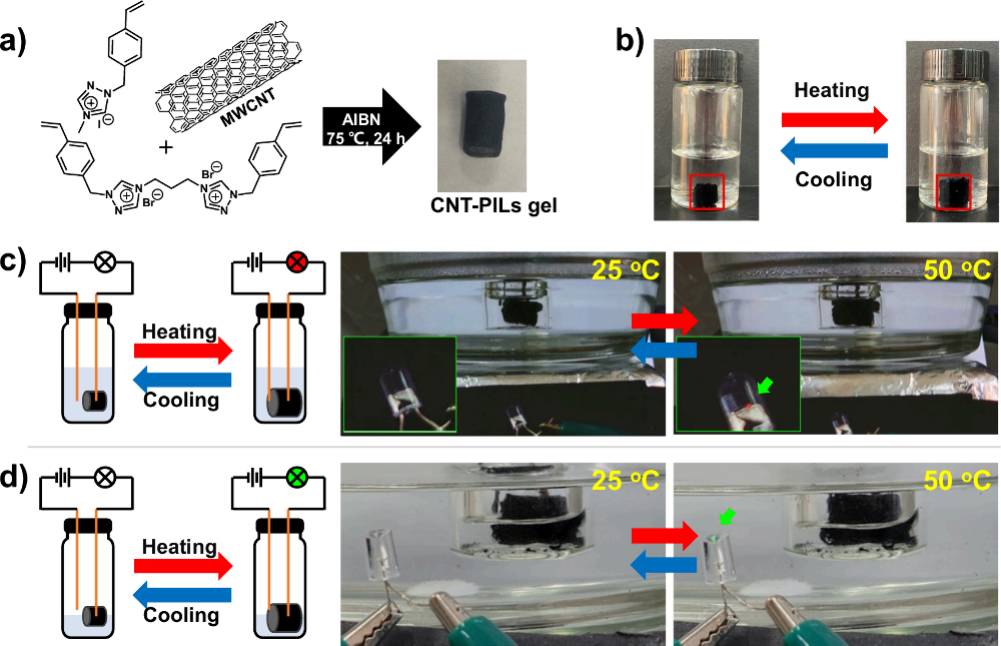
(a) Schematic representation of the synthesis of the CNT-PILs organogel. (b) Swelling and deswelling of the CNT-PILs organogel in methanol along the temperature change. (c and d) Schematic diagram of the circuit connection with the CNT-PILs organogel as a switch. The corresponding complete circuit is composed of an LED bulb and a CNT-PILs organogel.

Finally, we applied such thermosensitive organogels to “smart” switches. Due to the poor electron conductivity of the Ptriaz-C1-I gel containing a large amount of methanol, we doped the gel with CNTs to obtain a CNT-PIL composite gel (Figure 5a) with better conductivity. The CNT-PILs gel was found to exhibit the same UCST-type phase transition with methanol as the solvent (Figure 5b). We connected the CNT-PILs gel in series to the circuit and investigated the temperature control of the circuit connectivity. Two cases were examined where the wire was immersed in methanol (Figure 5c) or not in contact with methanol (Figure 5d). It was found that, in either case, the CNT-PILs gel was too small to contact the left wire at 25 °C, and because the methanol solution was essentially nonconductive, the circuit was in an open state and the light-emitting-diode (LED) bulb remained dark. As the temperature rose, the CNT-PILs gel continued to swell until it touched the left wire, connecting the circuit and lighting up the LED bulb. The whole process can be seen in Videos S1 and S2. This thermoresponsive conductive gel is expected to be used as “smart” switches or protection from circuit overheating.

## 4 Conclusions

In summary, a series of 1,2,4-triazolium-based PILs were successfully synthesized, and the effect of the chemical structure on their thermal responsiveness was investigated. It was found that Ptriaz-C1-I and Ptriaz-C4-Br exhibited UCST-type phase transitions in methanol and isopropyl alcohol, respectively, and Ptriaz-C1-PF_6_ exhibited LCST-type phase transition in acetone. The thermal responsiveness is reversible and concentration-dependent. The temperature-variable ^1^H NMR results indicate that the UCST behavior of Ptriaz-C1-I in methanol is mainly caused by temperature-dependent changes in the solubility of the polymer backbone in methanol. Finally, the thermosensitive organogels with Ptriaz-C1-I structure were prepared and applied to the treatment of anion-containing organic waste liquids and temperature-controlled “smart” switches. Because the 1,2,4-triazolium-based thermoresponsive PILs are very unexplored, this study will inspire and guide the interest of the community in exploring this area.

## Supporting Information Available

Supplementary experimental procedures and characterization data (^1^H NMR, ^13^C NMR, HRMS, and results of molecular weight and anion exchange degree of PILs, FT-IR, DLS, and UV–vis) (PDF)
Video S1 (MP4)
Video S2 (MP4)

## References

[ref1] Hu L.; Wan Y.; Zhang Q.; Serpe M. J. Harnessing the Power of Stimuli-Responsive Polymers for Actuation. Adv. Funct. Mater. 2020, 30, 1903471 10.1002/adfm.201903471.

[ref2] Wei M.; Gao Y.; Li X.; Serpe M. J. Stimuli-Responsive Polymers and Their Applications. Polym. Chem. 2017, 8, 127–143. 10.1039/C6PY01585A.

[ref3] Zhao C.; Ma Z.; Zhu X. X. Rational Design of Thermoresponsive Polymers in Aqueous Solutions: A Thermodynamics Map. Prog. Polym. Sci. 2019, 90, 269–291. 10.1016/j.progpolymsci.2019.01.001.

[ref4] Luo G.-F.; Chen W.-H.; Zhang X.-Z. Poly(N-Isopropylacrylamide)-Based Thermally Responsive Micelles. ACS Macro Lett. 2020, 9, 872–881. 10.1021/acsmacrolett.0c00342. 35648534

[ref5] Zhou Q.; Men Y. Thermoresponsive Ionogels. Polym. Chem. 2024, 15, 2719–2739. 10.1039/D4PY00430B.

[ref6] Concilio M.; Beyer V. P.; Becer C. R. Thermoresponsive Polymers in Non-Aqueous Solutions. Polym. Chem. 2022, 13, 6423–6474. 10.1039/D2PY01147F.

[ref7] Scarpa J. S.; Mueller D. D.; Klotz I. M. Slow Hydrogen-Deuterium Exchange in a Non-.Alpha.-Helical Polyamide. J. Am. Chem. Soc. 1967, 89, 6024–6030. 10.1021/ja01000a006.

[ref8] Halperin A.; Kröger M.; Winnik F. M. Poly(N-Isopropylacrylamide) Phase Diagrams: Fifty Years of Research. Angew. Chem., Int. Ed. 2015, 54, 15342–15367. 10.1002/anie.201506663. 26612195

[ref9] Zhang S.-Y.; Zhuang Q.; Zhang M.; Wang H.; Gao Z.; Sun J.-K.; Yuan J. Poly(Ionic Liquid) Composites. Chem. Soc. Rev. 2020, 49, 1726–1755. 10.1039/C8CS00938D. 32096815

[ref10] Hu H.; Wang B.; Chen B.; Deng X.; Gao G. Swellable Poly(Ionic Liquid)s: Synthesis, Structure-Property Relationships and Applications. Prog. Polym. Sci. 2022, 134, 101607 10.1016/j.progpolymsci.2022.101607.

[ref11] Zhu M.; Yang Y. Poly(Ionic Liquid)S: An Emerging Platform for Green Chemistry. Green Chem. 2024, 26, 5022–5102. 10.1039/D4GC00202D.

[ref12] Kohno Y.; Saita S.; Men Y.; Yuan J.; Ohno H. Thermoresponsive Polyelectrolytes Derived from Ionic Liquids. Polym. Chem. 2015, 6, 2163–2178. 10.1039/C4PY01665C.

[ref13] Kohno Y.; Ohno H. Key Factors to Prepare Polyelectrolytes Showing Temperature-Sensitive Lower Critical Solution Temperature-Type Phase Transitions in Water. Aust. J. Chem. 2012, 65, 91 10.1071/CH11378.

[ref14] Men Y.; Li X.-H.; Antonietti M.; Yuan J. Poly(Tetrabutylphosphonium 4-Styrenesulfonate): A Poly(Ionic Liquid) Stabilizer for Graphene Being Multi-Responsive. Polym. Chem. 2012, 3, 871–873. 10.1039/c2py20011b.

[ref15] Zheng H.; Lv X.; Zhang Y.; Men Y. Enhancing Mechanical Strength in Thermosensitive Poly(Tetrabutylphosphonium Styrene Sulfonate) (PTPSS) Hydrogels through Acrylamide (AAM) Copolymerization. Polymer 2024, 308, 127420 10.1016/j.polymer.2024.127420.

[ref16] Seno K.-I.; Kanaoka S.; Aoshima S. Synthesis and Lcst-Type Phase Separation Behavior in Organic Solvents of Poly(Vinyl Ethers) with Pendant Imidazolium or Pyridinium Salts. J. Polym. Sci., Part A: Polym. Chem. 2008, 46, 5724–5733. 10.1002/pola.22881.

[ref17] Liu S.-h.; Wang H.; Sun J.-k.; Antonietti M.; Yuan J. Smart Hydrogen Atoms in Heterocyclic Cations of 1,2,4-Triazolium-Type Poly(Ionic Liquid)s. Acc. Chem. Res. 2022, 55, 3675–3687. 10.1021/acs.accounts.2c00430. 36469417 PMC9774662

[ref18] Jourdain A.; Asbai R.; Anaya O.; Chehimi M. M.; Drockenmuller E.; Montarnal D. Rheological Properties of Covalent Adaptable Networks with 1,2,3-Triazolium Cross-Links: The Missing Link between Vitrimers and Dissociative Networks. Macromolecules 2020, 53, 1884–1900. 10.1021/acs.macromol.9b02204.

[ref19] Akacha R.; Abdelhedi-Miladi I.; Serghei A.; Ben Romdhane H.; Drockenmuller E. 1,3,4,5-Tetrasubstituted Poly(1,2,3-Triazolium) Obtained through Metal-Free AA+BB Polyaddition of a Diazide and an Activated Internal Dialkyne. Macromol. Rapid Commun. 2024, 45, 2300644 10.1002/marc.202300644. 38350089

[ref20] Miller R. J.; Smith V. M.; Love S. A.; Byron S. M.; la Cruz D. S.-d.; Miller K. M. Synthesis and Evaluation of Cellulose-Based, 1,2,3-Triazolium-Functionalized Polymerized Ionic Liquids: Thermal Transitions, Ionic Conductivities, and Morphological Properties. ACS Appl. Polym. Mater. 2021, 3, 1097–1106. 10.1021/acsapm.0c01327.

[ref21] Hays E. A.; Eicher G.; Morales A.; la Cruz D. S.-d.; Miller K. M. Dual Ionic Liquid-Functionalized Cellulosic Materials: Thermal, Conductive, and Morphological Properties. ACS Appl. Polym. Mater. 2024, 6, 2616–2625. 10.1021/acsapm.3c02815.

[ref22] Cao W.; Tan L.; Wang H.; Yuan J. Dual-Cationic Poly(Ionic Liquid)s Carrying 1,2,4-Triazolium and Imidazolium Moieties: Synthesis and Formation of a Single-Component Porous Membrane. ACS Macro Lett. 2021, 10, 161–166. 10.1021/acsmacrolett.0c00784. 33489467 PMC7818656

[ref23] Zhang S.-Y.; Miao H.; Zhang H.-m.; Zhou J.-H.; Zhuang Q.; Zeng Y.-J.; Gao Z.; Yuan J.; Sun J.-K. Accelerating Crystallization of Open Organic Materials by Poly(Ionic Liquid)s. Angew. Chem., Int. Ed. 2020, 59, 22109–22116. 10.1002/anie.202008415. PMC7756458 32748542

[ref24] Li M.; Xiao J.; Ge C.; Ling Y.; Tang H. Preparation and Thermoresponsive Properties of UCST-Type Glycopolypeptide Bearing Mannose Pendants and 3-Methyl-1,2,3-Triazolium Linkages in Ethanol or Ethanol/Water Solvent Mixtures. Colloid Polym. Sci. 2017, 295, 773–782. 10.1007/s00396-017-4064-2.

[ref25] Lee S. H.; Kim M. J.; Lee S.-H.; Kim J.; Park H.-J.; Lee J. Thiazolylmethyl Ortho-Substituted Phenyl Glucoside Library as Novel C-Aryl Glucoside SGLT2 Inhibitors. Eur. J. Med. Chem. 2011, 46, 2662–2675. 10.1016/j.ejmech.2011.03.052. 21514014

[ref26] Liu D.-C.; Zhang H.-J.; Jin C.-M.; Quan Z.-S. Synthesis and Biological Evaluation of Novel Benzothiazole Derivatives as Potential Anticonvulsant Agents. Molecules 2016, 21, 164 10.3390/molecules21020164. 26938519 PMC6274423

[ref27] Mirzaei Y. R.; Xue H.; Shreeve J. n. M. Low Melting N-4-Functionalized-1-Alkyl or Polyfluoroalkyl-1,2,4-Triazolium Salts. Inorg. Chem. 2004, 43, 361–367. 10.1021/ic0351693. 14704088

[ref28] Daily L. A.; Miller K. M. Correlating Structure with Thermal Properties for a Series of 1-Alkyl-4-Methyl-1,2,4-Triazolium Ionic Liquids. J. Org. Chem. 2013, 78, 4196–4201. 10.1021/jo4003932. 23530931

[ref29] Zhang W.; Kochovski Z.; Lu Y.; Schmidt B. V. K. J.; Antonietti M.; Yuan J. Internal Morphology-Controllable Self-Assembly in Poly(Ionic Liquid) Nanoparticles. ACS Nano 2016, 10, 7731–7737. 10.1021/acsnano.6b03135. 27501014

[ref30] Zhang W.; Yuan J. Poly(1-Vinyl-1,2,4-Triazolium) Poly(Ionic Liquid)s: Synthesis and the Unique Behavior in Loading Metal Ions. Macromol. Rapid Commun. 2016, 37, 1124–1129. 10.1002/marc.201600001. 26987872

[ref31] He H.; Luebke D.; Nulwala H.; Matyjaszewski K. Synthesis of Poly(Ionic Liquid)s by Atom Transfer Radical Polymerization with ppm of Cu Catalyst. Macromolecules 2014, 47, 6601–6609. 10.1021/ma501487u.

[ref32] Tang H.; Tang J.; Ding S.; Radosz M.; Shen Y. Atom Transfer Radical Polymerization of Styrenic Ionic Liquid Monomers and Carbon Dioxide Absorption of the Polymerized Ionic Liquids. J. Polym. Sci., Part A: Polym. Chem. 2005, 43, 1432–1443. 10.1002/pola.20600.

[ref33] Yahia M.; Mei S.; Mathew A. P.; Yuan J. Linear Main-Chain 1,2,4-Triazolium Poly(Ionic Liquid)s: Single-Step Synthesis and Stabilization of Cellulose Nanocrystals. ACS Macro Lett. 2019, 8, 1372–1377. 10.1021/acsmacrolett.9b00542. 35651167

[ref34] Li M.; Xu Y.; Liu T.; Li Y.; Ling Y.; Tang H. Preparation and Thermoresponsive Properties of UCST-Type Polypeptide Bearing P-Tolyl Pendants and 3-Methyl-1,2,3-Triazolium Linkages in Methanol or Ethanol/Water Solvent Mixtures. Macromol. Chem. Phys. 2017, 218, 1700006 10.1002/macp.201700006.

[ref35] Cheng W.; Chen X.; Sun J.; Wang J.; Zhang S. SBA-15 Supported Triazolium-Based Ionic Liquids as Highly Efficient and Recyclable Catalysts for Fixation of CO2 with Epoxides. Catal. Today 2013, 200, 117–124. 10.1016/j.cattod.2012.10.001.

[ref36] S A.; M S. [Emim] BF4 Ionic Liquid-Mediated Synthesis of TiO2 Nanoparticles Using Vitex Negundo Linn Extract and Its Antibacterial Activity. J. Mol. Liq. 2016, 221, 986–992. 10.1016/j.molliq.2016.06.079.

[ref37] Mecerreyes D. Polymeric Ionic Liquids: Broadening the Properties and Applications of Polyelectrolytes. Prog. Polym. Sci. 2011, 36, 1629–1648. 10.1016/j.progpolymsci.2011.05.007.

[ref38] Robinson A. L. The Boiling-Point Elevation of Acetone Solutions as Related to the Interionic Attraction Theory. J. Phys. Chem. 1929, 33, 1193–1199. 10.1021/j150302a009.

[ref39] Sun J. K.; Zhang W.; Guterman R.; Lin H.; Yuan J. Porous Polycarbene-Bearing Membrane Actuator for Ultrasensitive Weak-Acid Detection and Real-Time Chemical Reaction Monitoring. Nat. Commun. 2018, 9. 10.1038/s41467-018-03938-x PMC5928224 29712899

[ref40] Li W.; Wu P. Unusual Thermal Phase Transition Behavior of an Ionic Liquid and Poly(Ionic Liquid) in Water with Significantly Different LCST and Dynamic Mechanism. Polym. Chem. 2014, 5, 5578–5590. 10.1039/C4PY00593G.

[ref41] Wang G.; Wu P. In-Depth Study of the Phase Separation Behaviour of a Thermoresponsive Ionic Liquid and a Poly(Ionic Liquid) in Concentrated Aqueous Solution. Soft Matter 2015, 11, 5253–5264. 10.1039/C5SM00603A. 26052832

[ref42] Kuzina M. A.; Kartsev D. D.; Stratonovich A. V.; Levkin P. A. Organogels Versus Hydrogels: Advantages, Challenges, and Applications. Adv. Funct. Mater. 2023, 33, 2301421 10.1002/adfm.202301421.

[ref43] Urata C.; Nagashima H.; Hatton B. D.; Hozumi A. Transparent Organogel Films Showing Extremely Efficient and Durable Anti-Icing Performance. ACS Appl. Mater. Interfaces 2021, 13, 28925–28937. 10.1021/acsami.1c06815. 34121387

[ref44] Zhuo Y.; Chen J.; Xiao S.; Li T.; Wang F.; He J.; Zhang Z. Gels as Emerging Anti-Icing Materials: A Mini Review. Mater. Horiz. 2021, 8, 3266–3280. 10.1039/D1MH00910A. 34842262

[ref45] Lv J.; Yao X.; Zheng Y.; Wang J.; Jiang L. Antiadhesion Organogel Materials: From Liquid to Solid. Adv. Mater. 2017, 29, 1703032 10.1002/adma.201703032. 29058798

