# Supplementary material for: Thermal Responsiveness of 1,2,4-Triazolium-Based Poly(ionic liquid)s and Their Applications in Dye Extraction and Smart Switch
Source: ACS Appl Polym Mater. 2024 Oct 28;6(21):13202–9. doi: 10.1021/acsapm.4c02446 (PMC11555636; doi:10.1021/acsapm.4c02446)
Supplement: Supplementary file 1 — ap4c02446_si_001.pdf [file ap4c02446_si_001.pdf]

Supporting information

Thermal responsiveness of 1,2,4-triazolium-based poly(ionic liquid)s and their applications in dye extraction and smart switch

Feng Chen,[1,2,#] Jiefeng Zhu,[1,#] Ruijie Hou,[1] Xianjing Zhou,[1,*] Jiayin Yuan,[3,*] Xinping Wang[1]

[1] School of Chemistry and Chemical Engineering, Key Laboratory of Surface & Interface Science of Polymer Materials of Zhejiang Province, Zhejiang Sci-Tech University, Hangzhou 310018, China

[2] State Key Laboratory for Modification of Chemical Fibers and Polymer Materials, College of Materials Science and Engineering, Donghua University, Shanghai 201620, China

[3] Department of Materials and Environmental Chemistry, Stockholm University, Stockholm 10691, Sweden

---

[#] These authors contributed equally to this work.

[*] Corresponding author.
Xianjing Zhou (E-mail: xjzhou@zstu.edu.cn) & Jiayin Yuan (E-mail: jiayin.yuan@mmk.su.se)

1. Experimental Section

1.1. Synthesis of Im-C1-Cl.

4-Vinylbenzyl chloride (4.43 g, 29 mmol, 1 eq) and 1-methylimidazole (2.47 g, 30 mmol, 1 eq) were dissolved in 10 mL of ethanol. Hydroquinone (10 mg) was added in the reaction mixture as polymerization inhibitor. The reaction mixture was then stirred at 45℃ in nitrogen atmosphere for 24 h. After the reaction the solution was concentrated in vacuum to remove ethanol. The product was precipitated in cold ether. Finally the product was dried under vacuum at room temperature to give a yellow oil (5.84 g, 85.9%).

1.2. Synthesis of PIm-C1-Cl.

Im-C1-Cl (5.84 g, 24.9 mmol, 1 eq) and AIBN (0.0584 g, 0.35 mmol, 0.014 eq) were dissolved in 10 mL of ethanol. The reaction mixture was then stirred at 75 °C under nitrogen for 24 h. The reaction mixture was precipitated in cold ether, filtered off and the residue was dried under vacuum at room temperature to give a yellow solid (3.57 g, 61%).

1.3. Synthesis of PIm-C1-I.

PIm-C1-Cl (1 g) was dissolved in 10 mL of water. Potassium iodide (KI, 20 g) was dissolved in 50 mL of water and the aqueous PIm-Cl solution was added. The above aqueous solutions were mixed and stirred for 24 h at room temperature. The mixture was purified by dialysis against water to remove small molecules. A white solid was obtained after freeze-drying.

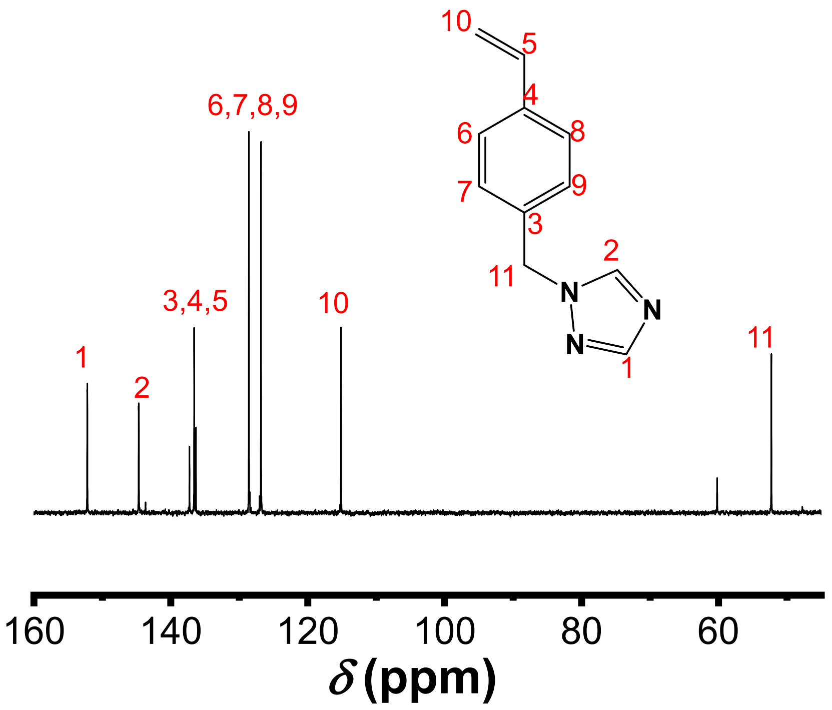

Figure S1. ^{13}C NMR spectrum of the neutral triaz monomer. The solvent is DMSO-d_6.

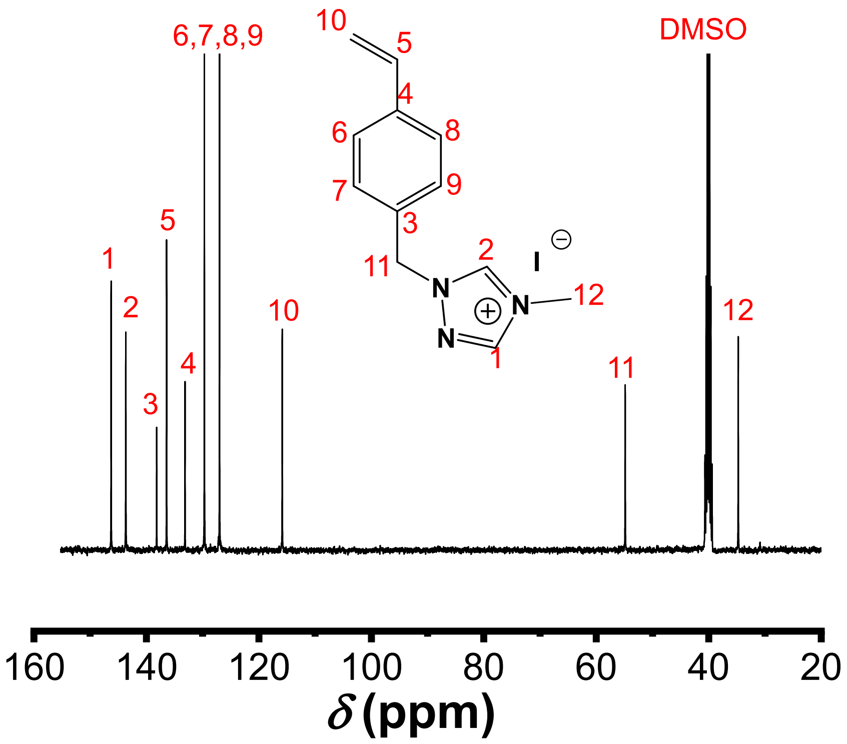

Figure S2. ^{13}C NMR spectrum of the triaz-C1-I monomer with I^- as anion. The solvent is DMSO-d_6.

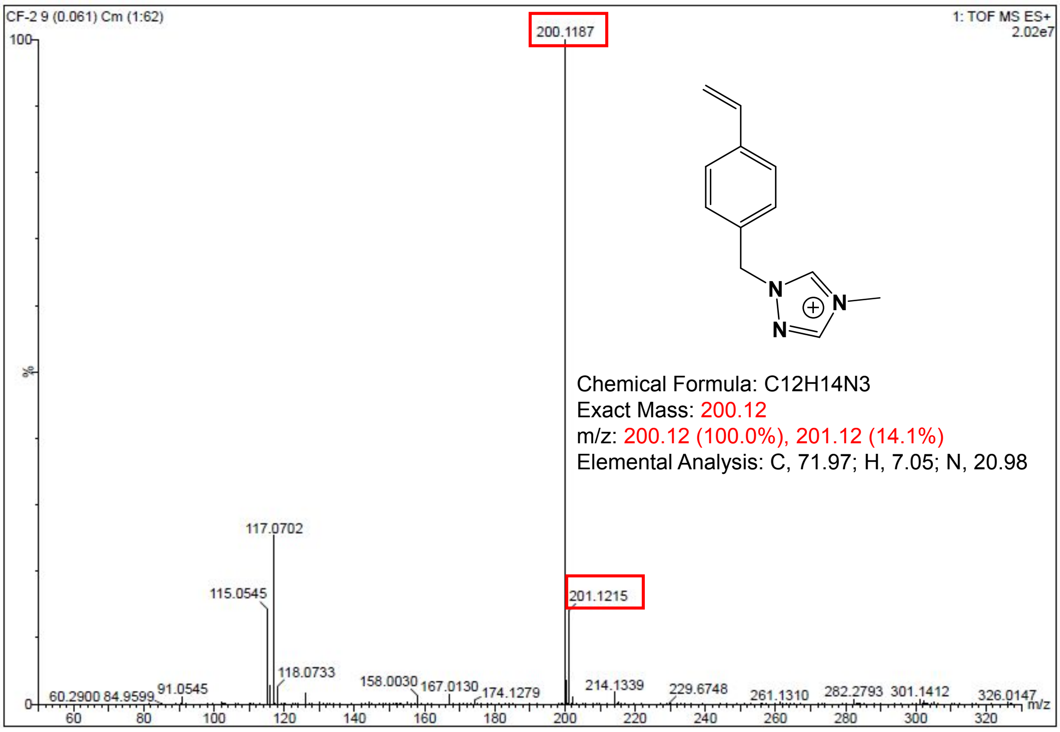

Figure S3. High resolution mass spectrum of the triaz-C1-I monomer with I^- as anion.

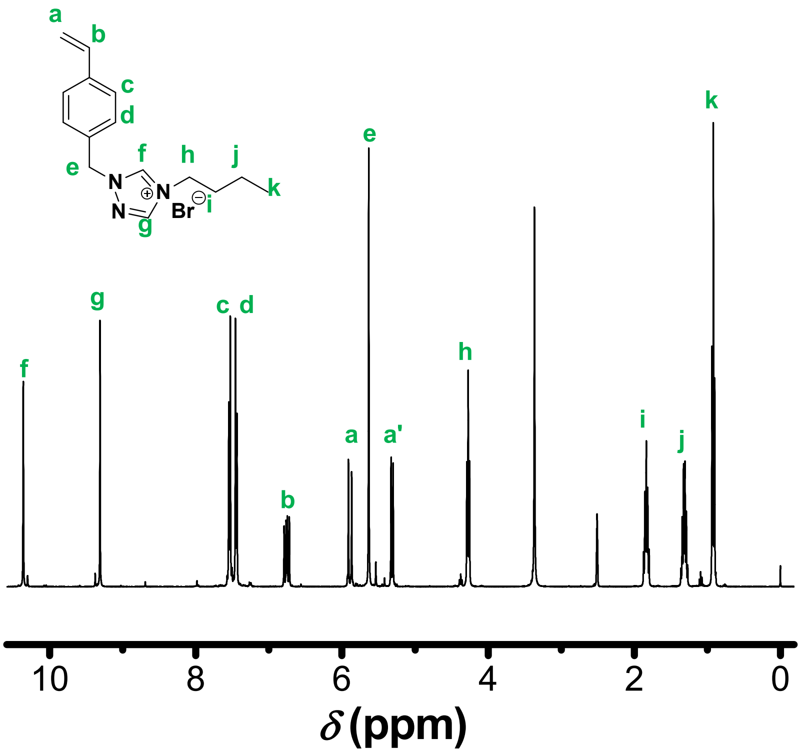

Figure S4. ^{1}H NMR spectrum of the triaz-C4-Br monomer with Br^- as anion. The solvent is DMSO-d_6.

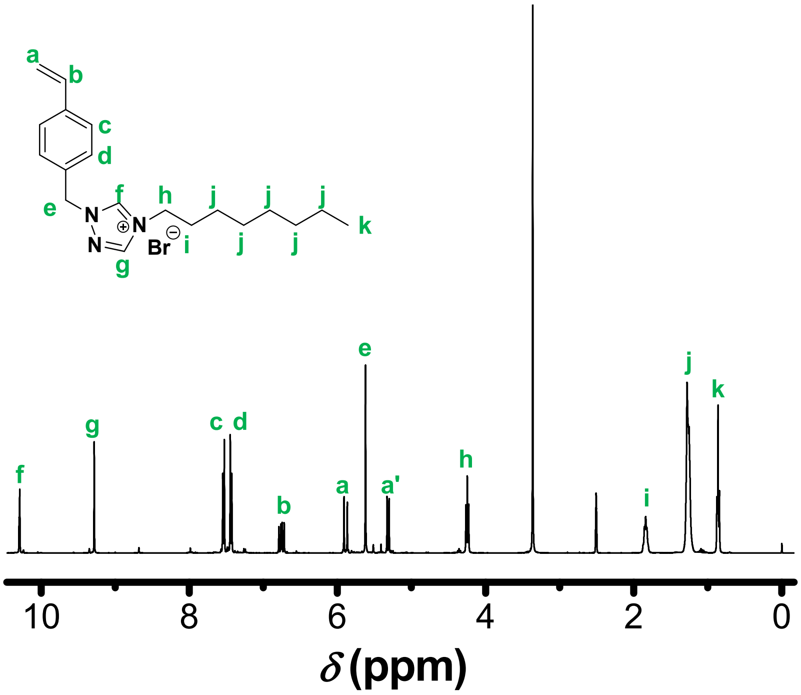

Figure S5. ^{1}H NMR spectrum of the triaz-C8-Br monomer with Br^- as anion. The solvent is DMSO-d_6.

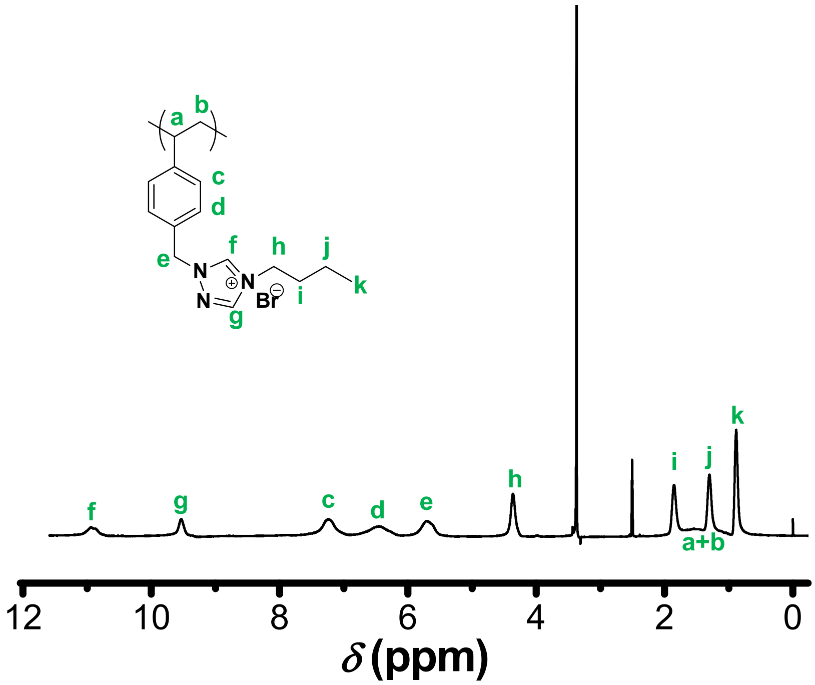

Figure S6. ^{1}H NMR spectrum of Ptriaz-C4-Br. The solvent is DMSO-d_6.

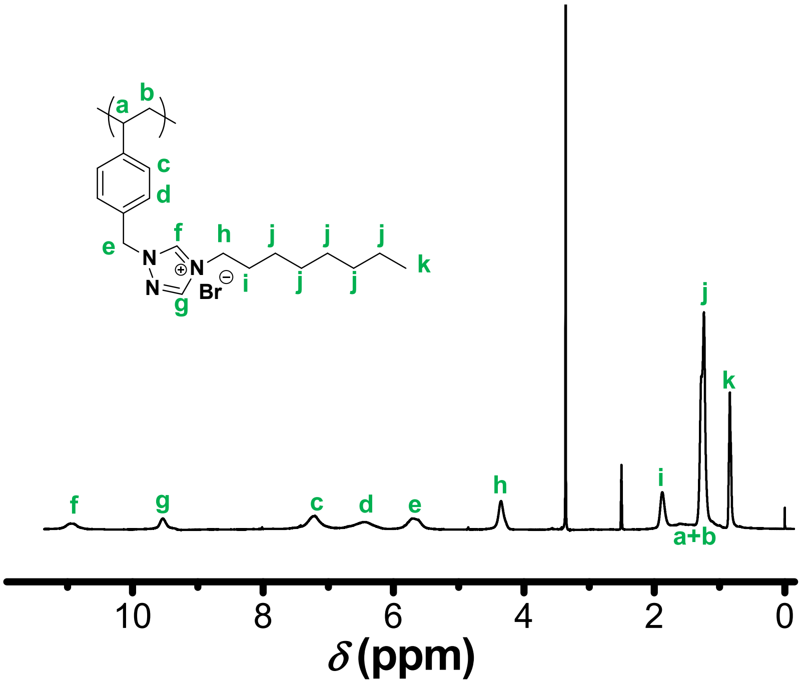

Figure S7. ^{1}H NMR spectrum of Ptriaz-C8-Br. The solvent is DMSO-d_6.

Table S1. Characterization of the molecular weight and the degree of anion exchange of different PIL samples.

PILs	M_n [a]	M_w [a]	$Đ$ [a]	Degree of anion exchange [c]
Ptriaz-C1-I	5600	15100	2.69	/
Ptriaz-C1-BF_4	/ [b]	/	/	84%
Ptriaz-C1-PF_6	/ [b]	/	/	95%
Ptriaz-C1-Br	/ [b]	/	/	80%
Ptriaz-C4-Br	4900	9100	1.84	/
Ptriaz-C8-Br	4800	9100	1.84	/

[a] Molecular weight and the polydispersity ($Đ$) of samples were determined by SEC using poly(methyl methacrylate) as standards.

[b] Ptriaz-C1-BF_4, Ptriaz-C1-PF_6, and Ptriaz-C1-Br are obtained by anion-exchange reaction of Ptriaz-C1-I, and their molecular weight information can be referred to Ptriaz-C1-I.

[c] The total organic halogen analyzer (AOX/TOX multi X® 2500 analyzer) was used to assess the change in iodide ion content in PILs before and after anion exchange. The

degree of anion exchange was determined by comparing the exchanged iodide ion content to the original content in the polymer.

SEC traces of Ptriaz-C1-I, Ptriaz-C4-Br, and Ptriaz-C8-Br were shown as follows:

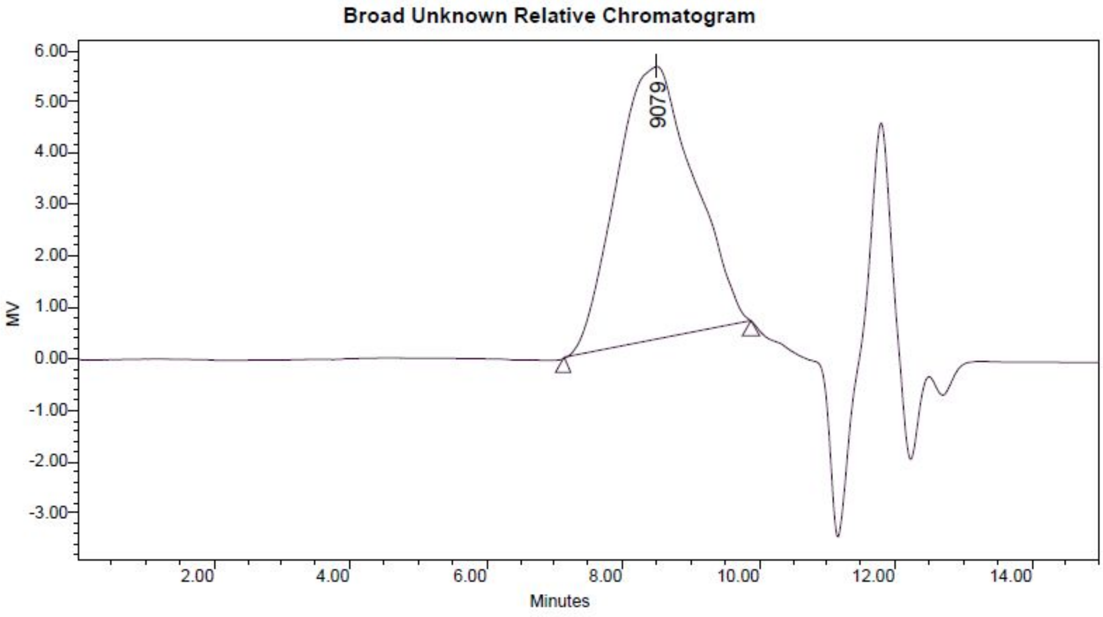

Broad Unknown Relative Peak Table

	Distribution Name	Mn (Daltons)	Mw (Daltons)	MP (Daltons)	Mz (Daltons)	Mz+1 (Daltons)	Polydispersity	Mz/Mw	Mz+1/Mw
1		5620	15165	9079	33554	55273	2.698546	2.212641	3.644801

Figure S8. SEC trace of Ptriaz-C1-I.

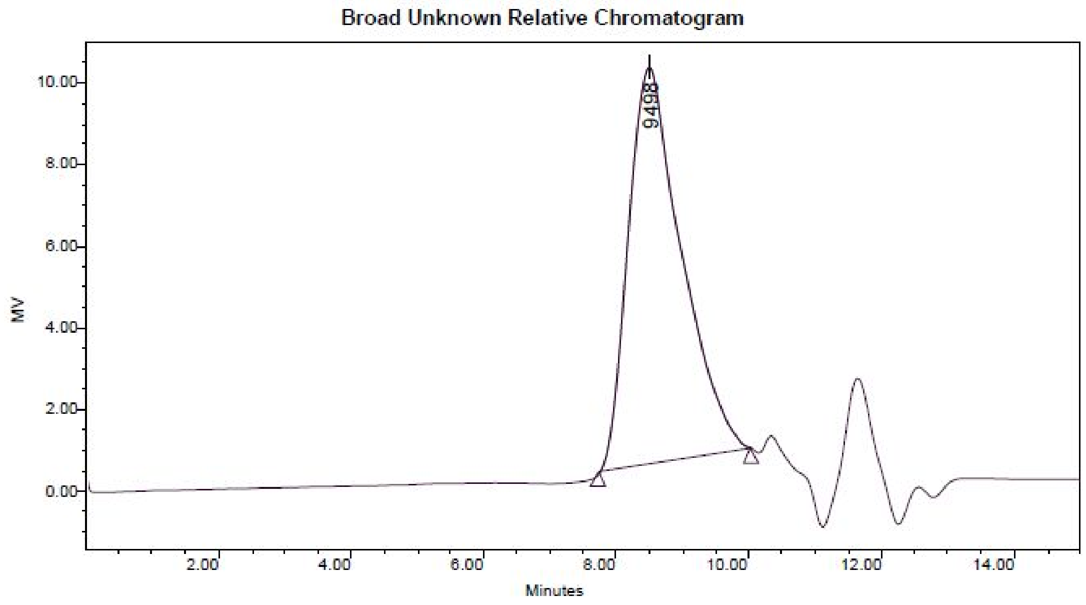

Broad Unknown Relative Peak Table

	Distribution Name	Mn (Daltons)	Mw (Daltons)	MP (Daltons)	Mz (Daltons)	Mz+1 (Daltons)	Polydispersity	Mz/Mw	Mz+1/Mw
1		4924	9061	9498	13504	17547	1.840183	1.490265	1.936526

Figure S9. SEC trace of Ptriaz-C4-Br.

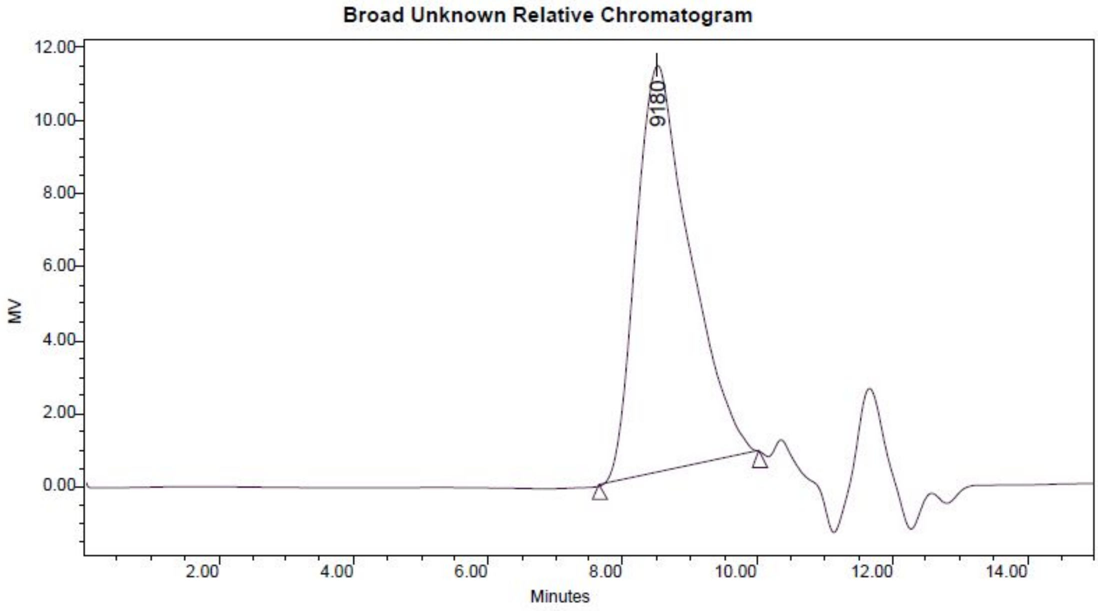

Broad Unknown Relative Peak Table

	Distribution Name	Mn (Daltons)	Mw (Daltons)	MP (Daltons)	Mz (Daltons)	Mz+1 (Daltons)	Polydispersity	Mz/Mw	Mz+1/Mw
1		4824	8858	9180	13324	17579	1.836248	1.504243	1.984557

Figure S10. SEC trace of Ptriaz-C8-Br.

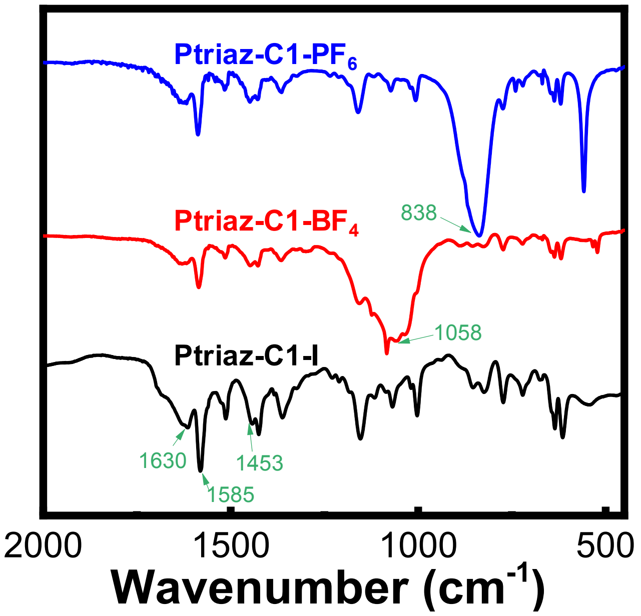

Figure S11. The FT-IR spectra of Ptriaz-C1-X (X = I, BF_4, or PF_6).

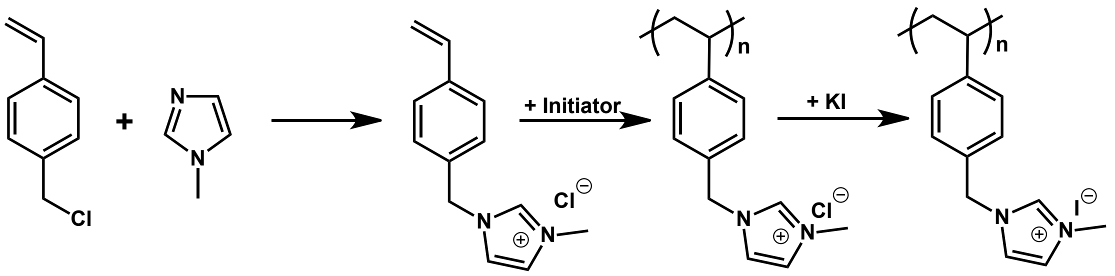

Figure S12 Synthetic route toward PIm-C1-I PIL.

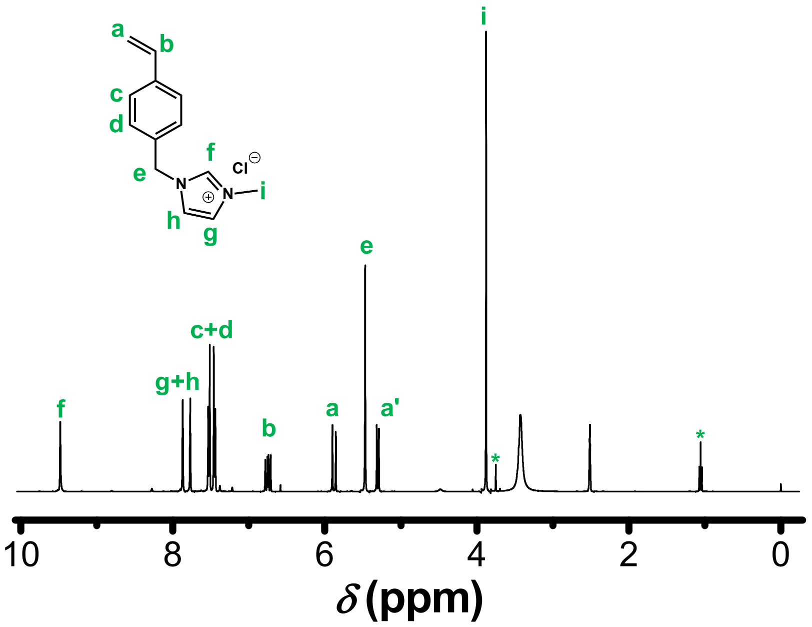

Figure S13 ^{1}H NMR spectrum of Im-C1-Cl. The solvent is DMSO-d_6. The marked region indicates solvent residue of ethanol.

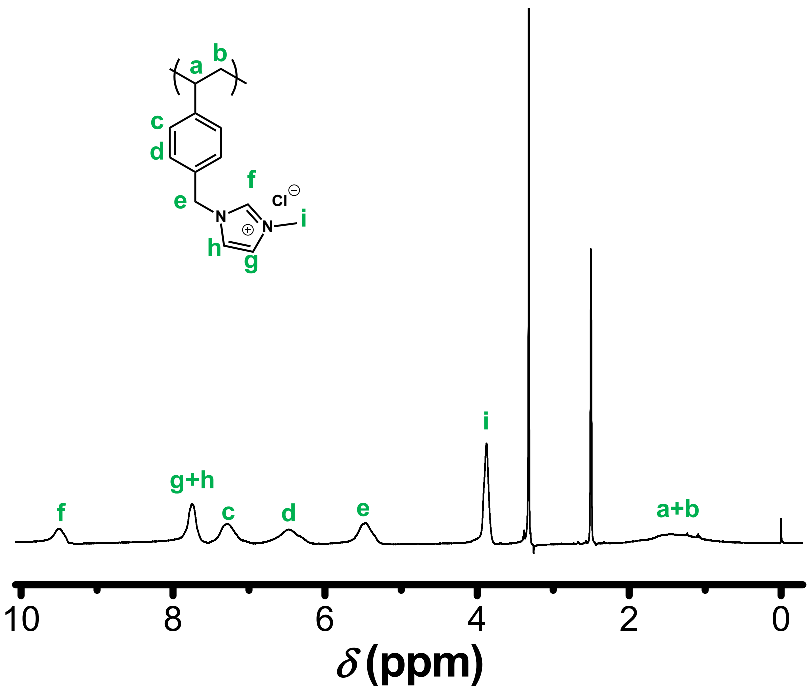

Figure S14 ^{1}H NMR spectrum of PIm-C1-Cl. The solvent is DMSO-d_6.

Table S2. Solubility table of PILs in various solvents at 25 and 80 °C.

PILs\solvent [a]	H_2O	MeOH	EtOH	acetone	THF	$CHCl_3$	toluene	DMSO	DMF	ACN	EA	IPA
**Ptriaz-C1-I**	+[b]	UCST	+/-	-	-	-	-	+	+	+/-	+/-	+/-
**Ptriaz-C1-BF_4**	+	+	+/-	+/-	-	+/-	-	+	+	+	+/-	+/-
**Ptriaz-C1-PF_6**	+/-	+/-	+/-	LCST	-	+/-	-	+	+	+	+/-	+/-
**PIm-C1-I**	+	+	+/-	+/-	-	-	-	+	+	+/-	-	+/-
**Ptriaz-C1-Br**	+	+	+/-	-	-	-	+/-	+	+	+/-	-	+/-
**Ptriaz-C4-Br**	+	+	+	-	-	+/-	+/-	+	+	+/-	-	UCST
**Ptriaz-C8-Br**	+/-	+	+	-	+/-	+	+/-	+	+	+	-	+/-

[a]Abbreviations of solvents: THF for tetrahydrofuran; DMSO for dimethyl sulfoxide; DMF for *N,N*-dimethylformamide; ACN for acetonitrile; EA for ethyl acetate; IPA for isopropanol. [b]+: soluble at 10 mg/mL; +/-: slightly soluble at 10 mg/mL; -: insoluble at 10 mg/mL.

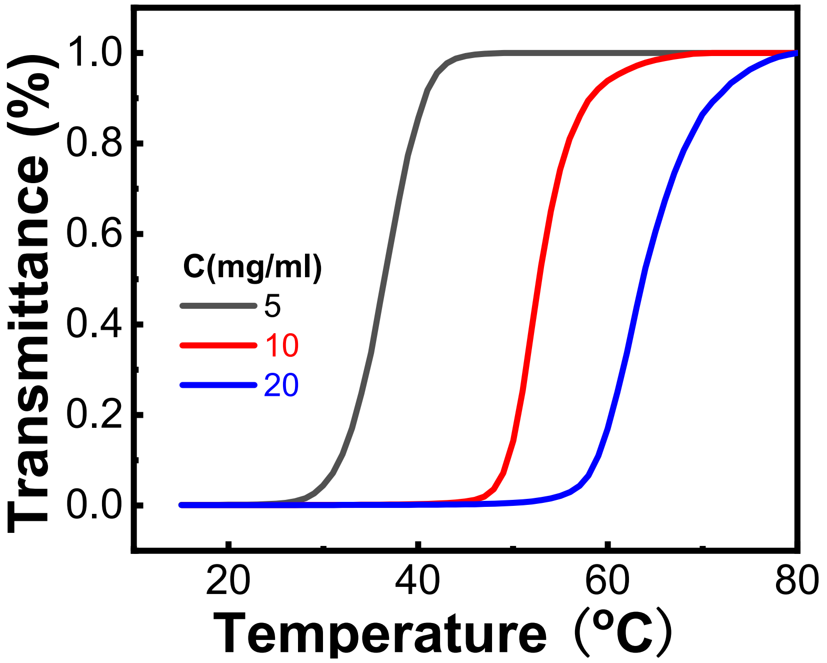

Figure S15. Turbidity curves of Ptriaz-C4-Br solution in isopropanol at various concentrations.

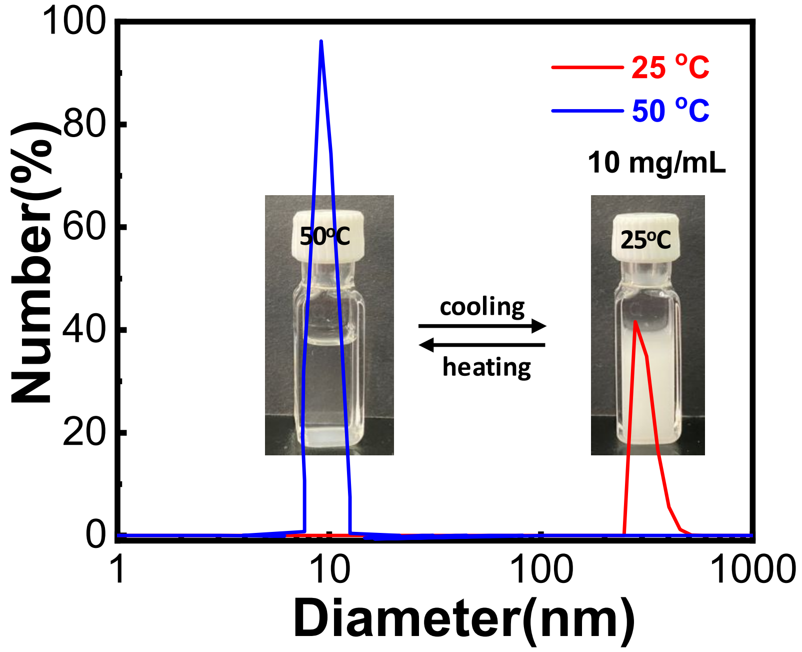

Figure S16. Hydrodynamic diameters of the Ptriaz-C4-Br solution in isopropanol below and above T_c. Insets are the photographs of the corresponding solutions.

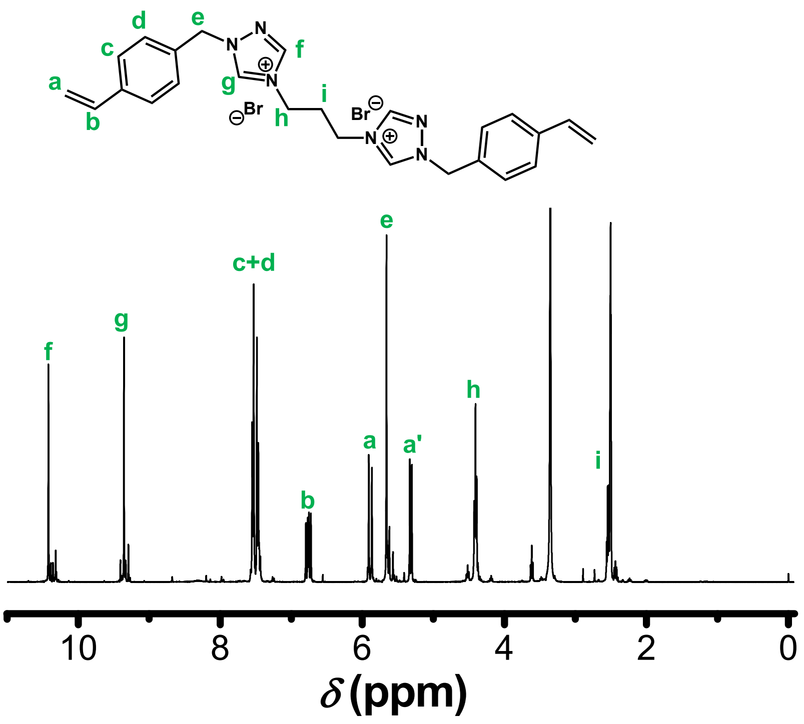

Figure S17. ^{1}H NMR spectrum of Dtriaz-Br. The solvent is DMSO-d_6.

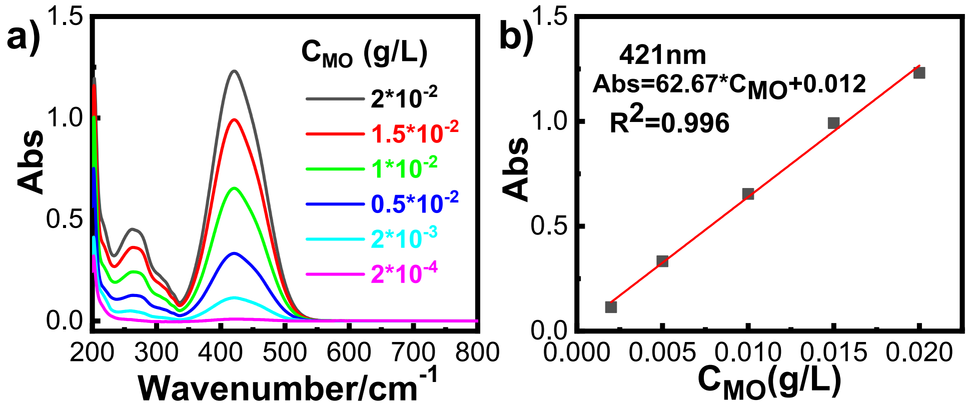

Figure S18. (a) UV-Vis absorption spectra and (b) the standard curves of methyl orange (MO) in methanol.

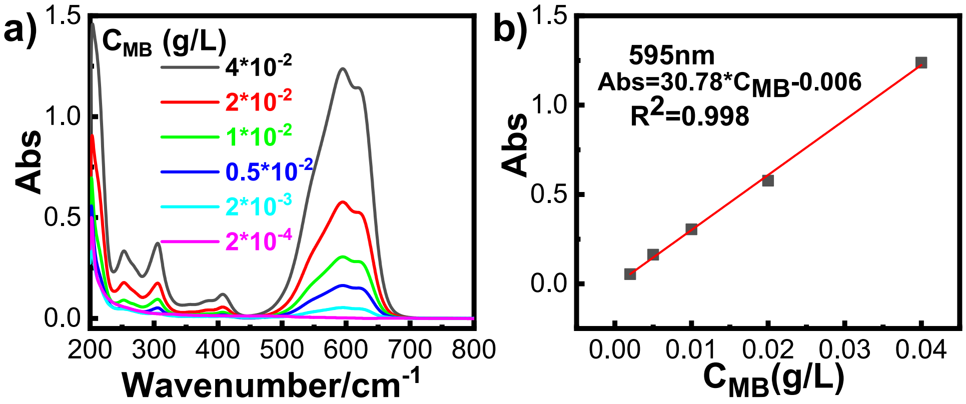

Figure S19. (a) UV-Vis absorption spectra and (b) standard curves of methyl blue (MB) in methanol.

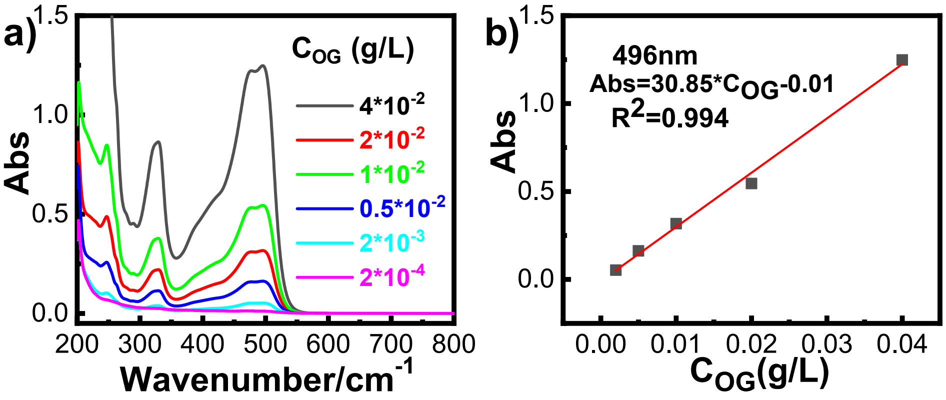

Figure S20. (a) UV-Vis absorption spectra and (b) standard curves of orange G (OG) in methanol.

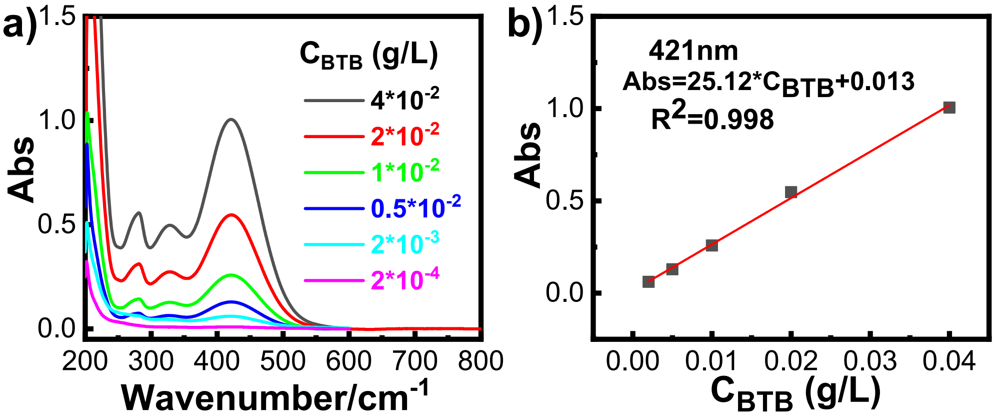

Figure S21 (a) UV-Vis absorption spectra and (b) standard curves of bromothymol

blue (BTB) in methanol.
